# The combination of cantharidin and antiangiogenic therapeutics presents additive antitumor effects against pancreatic cancer

**DOI:** 10.1038/s41389-018-0102-2

**Published:** 2018-11-26

**Authors:** Meng-Dan Xu, Lu Liu, Meng-Yao Wu, Min Jiang, Liu-Mei Shou, Wen-Jie Wang, Jing Wu, Yan Zhang, Fei-Ran Gong, Kai Chen, Min Tao, Qiaoming Zhi, Wei Li

**Affiliations:** 1 Department of Oncology, the First Affiliated Hospital of Soochow University, 215006 Suzhou, China; 2 Department of Emergency, the First Affiliated Hospital of Soochow University, 215006 Suzhou, China; 3 Department of Oncology, the First Affiliated Hospital of Zhejiang Chinese Medicine University, 310006 Hangzhou, China; 4 Department of Radio-Oncology, Nanjing Medical University Affiliated Suzhou Hospital, 215001 Suzhou, China; 5 Department of Hematology, the First Affiliated Hospital of Soochow University, 215006 Suzhou, China; 6 PREMED Key Laboratory for Precision Medicine, Soochow University, 215021 Suzhou, China; 7 Department of General Surgery, the First Affiliated Hospital of Soochow University, 215006 Suzhou, China; 8 Comprehensive Cancer Center, Suzhou Xiangcheng People’s Hospital, 215000 Suzhou, China

## Abstract

Cantharidin, one of the active components of mylabris, is believed to have antitumor activity. Cantharidin selectively inhibits protein phosphatase 2A (PP2A), which can repress multiple oncogenic kinases (ERK, JNK, PKC, and NF-κB). Researches in vitro have shown that cantharidin suppresses cell viability and metastasis in pancreatic cancer cells. This study aims to investigate the effects of cantharidin on pancreatic cancer xenografts in vivo. Xenograft models were established using cells stably expressing luciferase. Xenograft growth was evaluated by living imaging. Gene expression was determined using a microarray, real-time PCR, a RayBiotech antibody array, and the Milliplex assay. Surprisingly, cantharidin significantly accelerated xenograft growth. Living imaging showed a rapid distribution of D-luciferin in cantharidin-treated xenografts, suggesting a rich blood supply. Immunohistochemistry confirmed increased angiogenesis. Microarray and antibody array identified upregulated proangiogenic and downregulated antiangiogenic factors. The Milliplex assay suggested elevated secretion of IL-6, IL-8, TNF-α, and VEGF. Inhibitors of ERK, JNK, PKC, and NF-κB pathway attenuated the cantharidin-induced changes to proangiogenic gene expression. PKC pathway-inhibiting tamoxifen or antiangiogenic therapeutics, including Ginsenoside Rg3, bevacizumab, Apatinib, and Endostar, antagonized the proangiogenic effect of cantharidin or its derivatives. These regimens presented remarkable additive antitumor effects in vivo. Although cantharidin presents antitumor effects in vitro and has been applied in clinical practice, we revealed an unfavorable proangiogenic side effect. We recommend that the clinical application of cantharidin should be performed on the premise of antivascularization therapy.

## Introduction

Pancreatic cancer is a malignant disease, the mortality of which almost parallels its incidence^1^. Compared with the steady increase in the survival rate of most cancers, little progress has been made in pancreatic cancers. More than 50% of patients suffering from this disease are diagnosed at advanced or distant stages and are refractory to conventional treatments^2^. It is estimated that the current 5-year relative survival is 8% in the United States (2017) and continues to increase slightly (by 0.3% per year) in men^3^. Therefore, new strategies are urgently required to overcome this malignant disease.

Cantharidin is one of the active ingredients of mylabris. It is believed to have antitumor effect and has been widely used in China. Cantharidin selectively inhibits protein phosphatase 2A (PP2A), a repressor of several oncogenic kinase pathways, including extracellular signal-related kinase (ERK), c‑Jun N‑terminal kinase (JNK), protein kinase C (PKC), and nuclear factor kappa-light-chain-enhancer of activated B cells (NF-κB), all of which play important roles in controlling cell cycle, apoptosis, and determining cell fate^4^. Therefore, it is contradictory that cantharidin, an inhibitor of cancer-repressing PP2A, should present an antitumor effect. Our previous studies demonstrated that sustained activation of the JNK and NF-κB pathways, induced by PP2A inhibition, was responsible for the growth inhibition of cantharidin, indicating that activation of these kinase pathways was not always facilitating cancer progress. Moreover, cantharidin inhibited migration, arrested the G2/M cell cycle transition, induced apoptosis, repressed invasion, and impaired the stemness of pancreatic cancer cells in vitro^4–11^. However, these antitumor effects of cantharidin have not been verified in pancreatic cancer in vivo. So this study aimed to investigate the effect of cantharidin on pancreatic cancer xenografts in vivo.

## Results

### Cantharidin accelerated the growth of pancreatic cancer in both subcutaneous and orthotopic xenografts

**Fig. 1:**
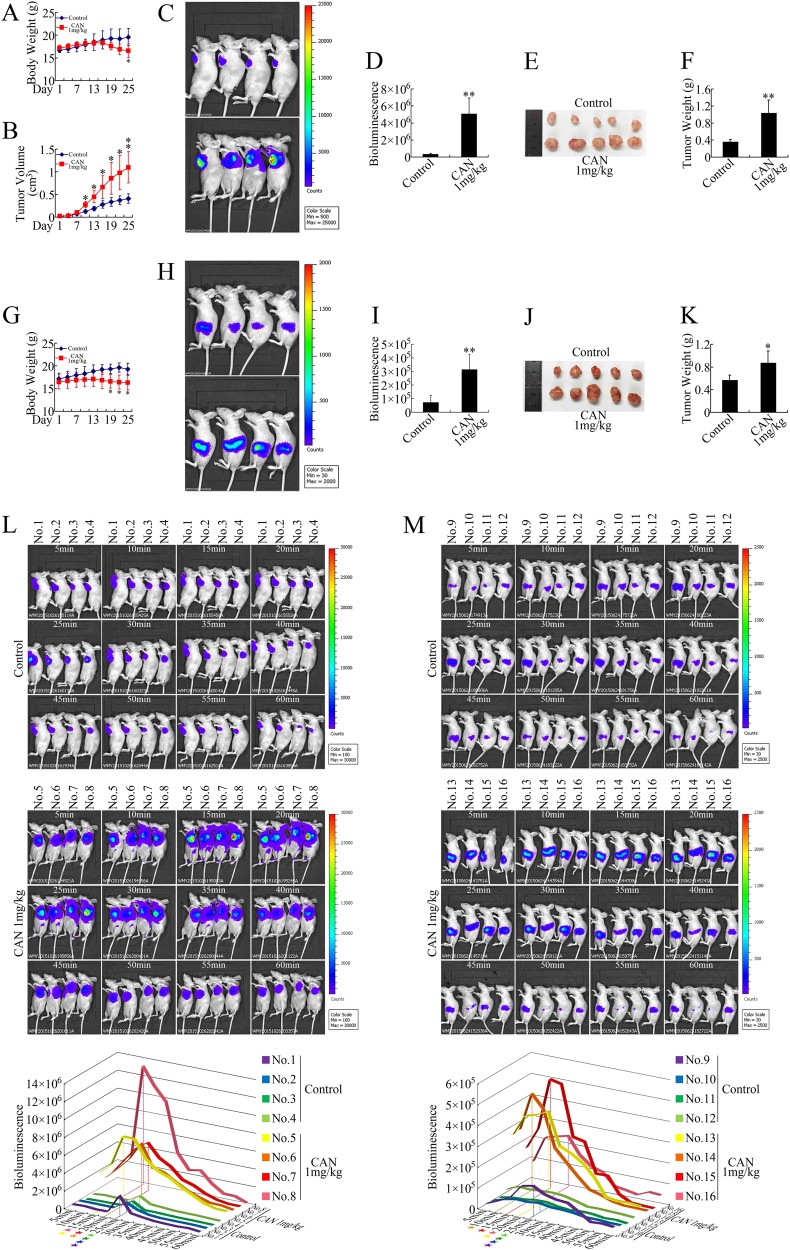
Cantharidin accelerated the growth of pancreatic cancer in both subcutaneous and orthotopic xenografts. PANC-1 cells were used in both subcutaneous and orthotopic pancreatic xenograft models. **a**, **b** Body weight (**a**) and tumor volume (**b**) evaluations of the cantharidin (CAN) group vs. the control group in subcutaneous xenografts. **c**, **d** Representative in vivo bioluminescent images (**c**) and bioluminescence analysis (**d**) of pancreatic cancer subcutaneous xenografts. **e**, **f** Photographs of subcutaneous xenograft (**e**) and tumor weight (**f**) in the CAN vs. the control group. **g** Body weight of the CAN vs. the control group in orthotopic xenografts. **h**, **i** Representative bioluminescent images (**h**) and bioluminescence analysis (**i**) of pancreatic cancer orthotopic xenografts. **j**, **k** Photographs of orthotopic xenograft (**j**) and tumor weight (**k**) in the CAN vs. the control group. **l**, **m** The bioluminescent images showing the process of bioluminescence peak emergence and decay in the CAN vs. the control group in subcutaneous (**l**) and orthotopic xenografts (**m**). *P < 0.05 and **P < 0.01, significant differences vs. the control group

As shown in Fig. 1a, b, surprisingly, the mice in the cantharidin-treated group showed significant body weight loss and enlarged tumor volumes compared with the control group. Living imaging showed that cantharidin significantly accelerated the growth of pancreatic cancer subcutaneous xenografts, rather than inhibiting them (Fig. 1c–f). Moreover, we identified similar results in lung cancer and colorectal cancer (Supplementary Fig. 1). Methyl thiazolyl tetrazolium (MTT) assays showed that cantharidin exhibited an inhibitory effect on the growth of both NCI-H292 lung cancer cells (Supplementary Fig. 1A) and LoVo colorectal cancer cells (Supplementary Fig. 1B) in vitro as well. However, in in vivo studies, cantharidin promoted the growth of NCI-H292 and LoVo xenografts (Supplementary Fig. 1C-F), suggesting that the pro-growth effect of cantharidin was independent of cancer type. To further confirm this confusing result, we established orthotopic xenograft models and found consistent results (Fig. 1g–k). Interestingly, by scanning the process using living imaging (Fig. 1l, m), we noticed that the bioluminescence peak value of the cantharidin-treated subjects emerged earlier than the control group. In addition, the bioluminescence of the cantharidin group also decayed faster, indicating a rapid distribution of D-luciferin. This phenomenon suggested that cantharidin-treated xenografts might have a rich blood supply, which led us to speculate that cantharidin might promote angiogenesis in vivo.

### Cantharidin promoted angiogenesis of a pancreatic cancer model

**Fig. 2:**
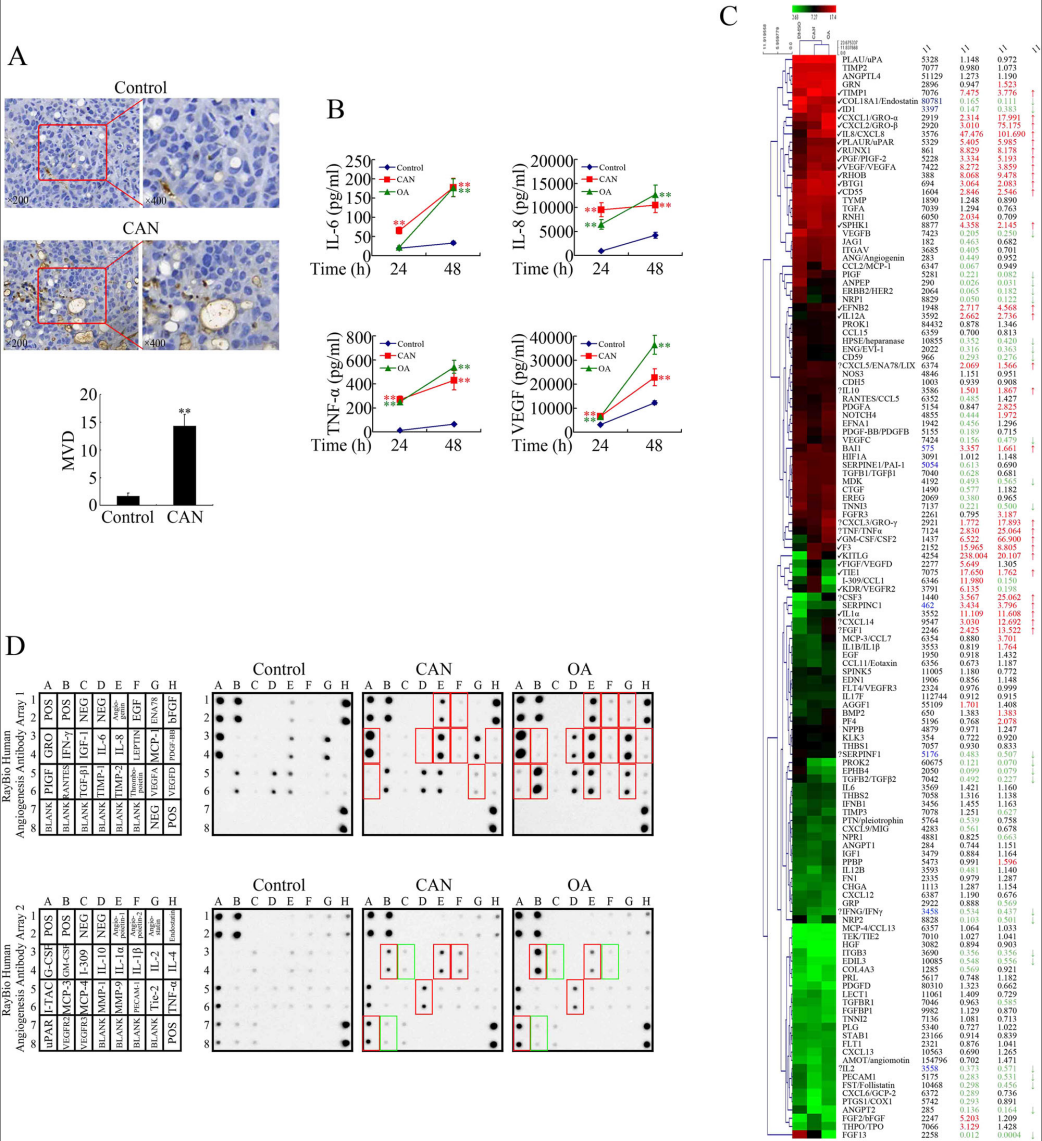
Cantharidin promoted angiogenesis of pancreatic cancer xenografts. **a** Immunohistochemical examination of pancreatic orthotopic xenografts targeting CD34 and microvessel density (MVD) evaluation. **b** Milliplex assay detecting proangiogenic factors in the medium after cantharidin (CAN) or okadaic acid (OA) treatments. *P < 0.05 and **P < 0.01, significant differences vs. control group. **c** Microarray analysis of angiogenesis-related gene expression levels upon CAN or OA treatments. Up and down arrows indicate gene expression levels that were significantly upregulated or downregulated by 1.5-fold. **d** Antibody arrays showing the protein expression of angiogenesis-related genes after CAN or OA treatments

To verify the hypothesis that cantharidin might promote angiogenesis, expression of CD34 was detected in cantharidin-treated pancreatic orthotopic xenograft specimens via immunohistochemistry. As shown in Fig. 2a, vascular endothelial cells were stained with CD34 antibody by immunohistochemistry. According to microvessel density (MVD) levels, we found that treatment with cantharidin promoted angiogenesis significantly compared with the control subjects. Similar results were obtained in xenografts of lung cancer (NCI-H292 cells) and colorectal cancer (LoVo cells), shown in Supplementary Fig. 2a, b, suggesting that the proangiogenic effect of cantharidin was independent of cancer type.

Angiogenesis is executed via activating multiply kinase pathways, including JNK, ERK, PKC, and NF-κB^12–15^. All these pathways share the same negative regulator, PP2A. Previously, we have proved that cantharidin could activate the pathways of ERK, JNK, PKC, and NF-κB through inhibition of PP2A^4–7,11^. Therefore, the proangiogenic effect of cantharidin could be related to PP2A inhibition. To verify this hypothesis, we assessed the secretion and expression of proangiogenic factors upon treatment with cantharidin and okadaic acid (OA), a classic PP2A inhibitor.

First, we used milliplex assays to measure the levels of proangiogenic interleukin (IL)-6, IL-8, tumor necrosis factor-α (TNF-α), and vascular endothelial growth factor (VEGF) in culture medium of PANC-1 cells upon treatment with cantharidin or OA. As expected, both cantharidin and OA time-dependently promoted the secretion of IL-6, IL-8, TNF-α, and VEGF (Fig. 2b).

Then we analyzed microarray data^5^ to determine the mRNA expression levels of angiogenesis-related genes upon treated with cantharidin or OA in PANC-1 cells. The microarray data are available from Gene Expression Omnibus (GEO) database, the accession code of which is GSE114288. As presented in Fig. 2c, both cantharidin and OA upregulated the expression levels of multiple proangiogenic genes, including *TIMP1*, *CXCL1/GRO-α*, *CXCL2/GRO-β*, *IL-8/CXCL8*, *PLAUR/uPAR*, *RUNX1*, *PGF/PIGF-2*, *VEGF/VEGFA*, *RHOB*, *BTG1*, *CD55*, *SPHK1*, *EFNB2*, *IL12A*, *CXCL5/ENA78/LIX*, *IL-10*, *BAI1*, *CXCL3/GRO-γ*, *TNF/TNF-α*, *GM-CSF/CSF2*, *F3*, *KITLG*, *TIE1*, *CSF3*, *SERPINC1*, *IL-1α*, *CXCL14*, and *FGF1*. The expression of antiangiogenic *COL18A1/Endostatin* was downregulated following cantharidin and OA treatments.

Furthermore, we assessed the protein levels of the angiogenesis-related genes after cantharidin or OA treatment using RayBio antibody arrays. Both cantharidin and OA upregulated the levels of provascularizing Angiogenin, epidermal growth factor EGF, GRO, IL-6, IL-8, LEPTIN, platelet-derived growth factor-BB, placenta growth factor, VEGFA, granulocyte macrophages colony-stimulating factor (GM-CSF), IL-1α, matrix metalloproteinase (MMP)-1, and urokinase-type plasminogen activator receptor (uPAR) (Fig. 2d). By cross-comparing the microarray datasets, we found that both cantharidin and OA could increase expression levels of *IL-8/CXCL8*, *CXCL1/GRO-α*, *VEGF/VEGFA*, *GM-CSF/CSF2*, and *PLAUR/uPAR* at both mRNA and protein levels. Therefore, the proangiogenic effect of cantharidin and OA may be executed through mechanisms involving upregulating multiple angiogenesis-related genes.

### ERK, JNK, PKC, and NF-κB kinase pathways participated in cantharidin-induced upregulation of proangiogenic genes

Cantharidin could activate ERK, JNK, PKC, and NF-κB pathways by inhibiting PP2A^4–7,11^; therefore, we discussed whether these kinase pathways participated in the upregulation process of proangiogenic genes upon treatment with cantharidin.

**Fig. 3:**
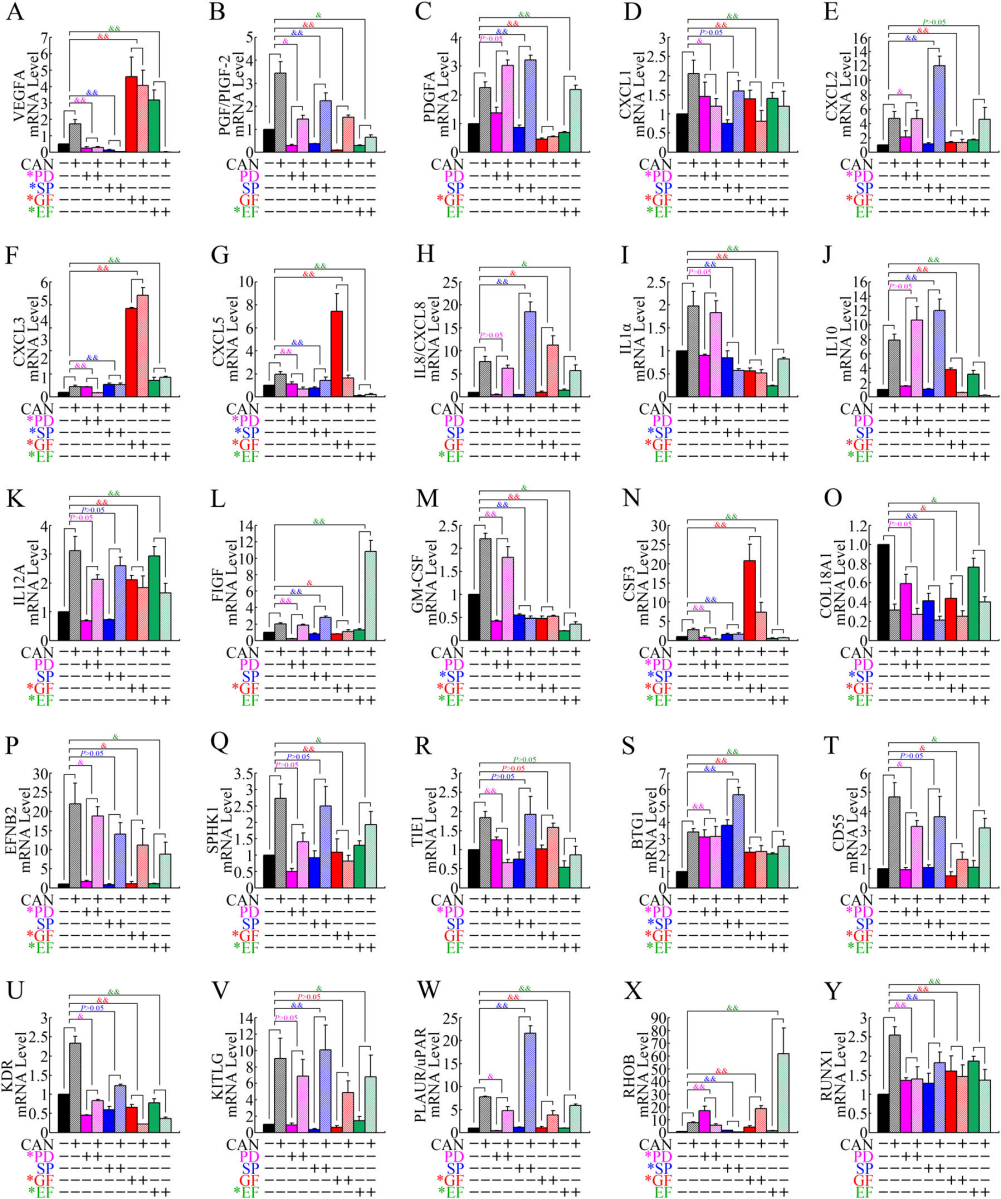
ERK, JNK, NF-κB, and PKC kinase pathways contributed to cantharidin-induced upregulation of proangiogenic genes. **a**–**y** Real-time PCR determination of the mRNA levels of angiogenesis-related genes, including *VEGFA* (**a**), *PGF/PIGF-2* (**b**), *PDGFA* (**c**), *CXCL1* (**d**), *CXCL2* (**e**), *CXCL3* (**f**), *CXCL5* (**g**), *IL-8/CXCL8* (**h**), *IL-1α* (**i**), *IL-10* (**j**), *IL12A* (**k**), *FIGF* (**l**), *GM-CSF* (**m**), *CSF3* (**n**), *COL18A1* (**o**), *EFNB2* (**p**), *SPHK1* (**q**), *TIE1* (**r**), *BTG1* (**s**), *CD55* (**t**), *KDR* (**u**), *KITLG* (**v**), *PLAUR/uPAR* (**w**), *RHOB* (**x**), and *RUNX1* (**y**), in PANC-1 cells after treatment with cantharidin (CAN) combined with ERK pathway inhibitor PD98059 (PD), JNK inhibitor SP600125 (SP), PKC inhibitor GF109203X (GF), or NF-κB inhibitor EF-24 (EF). ^&^*P* < 0.05 and ^&&^*P* < 0.01, significant differences between fold changes. Asterisk (*) indicates that the inhibitors attenuated the CAN-induced upregulation of the target genes

PANC-1 cells were pretreated with the ERK inhibitor PD98059, the JNK inhibitor SP600125, the NF-κB inhibitor EF-24, and the PKC inhibitor GF109203X, separately, followed by cantharidin treatment. We then detected the expression of the proangiogenic genes that were upregulated by cantharidin using real-time PCR. Finally, we discovered that inhibitors of ERK, JNK, PKC, and/or NF-κB attenuated the cantharidin-induced remodeling of proangiogenic gene expression profiles significantly (Fig. 3), suggesting that all these pathways contribute to the provascularizing effect of cantharidin. In addition, inhibitors of NF-κB and PKC pathways were involved in the upregulation of most of the target genes (Table 2), implying that these two kinase pathways might be the key pathways responsible for the upregulation of proangiogenic genes upon treatment with cantharidin.

**Table 2:** Summary of kinase pathways inhibitors attenuating the CAN-induced upregulation of proangiogenic genes

Genes	Kinase pathway inhibitors			
	PD98059	SP600125	GF109203X	EF-24
VEGFA	*	*	*	*
PGF/PIGF-2				*
PDGFA			*	
CXCL1	*		*	*
CXCL2	*		*	
CXCL3	*	*	*	*
CXCL5	*	*	*	*
IL-8/CXCL8				*
IL1α		*	*	
IL10			*	*
IL12A			*	*
FIGF			*	
GM-CSF		*	*	*
CSF3	*	*	*	*
COL18A1		*	*	*
EFNB2	*		*	*
SPHK1			*	*
TIE1	*			
BTG1	*	*	*	*
CD55	*		*	*
KDR	*		*	*
KITLG				*
PLAUR/uPAR			*	*
RHOB	*	*	*	
RUNX1				

Asterisk (*) indicates that the inhibitors attenuated the CAN-induced upregulation of the target genes

### Combination of cantharidin and Ginsenoside Rg3 or tamoxifen (TAM) presented additive anticancer effects

Cantharidin presented multiple antitumor effects in vitro; therefore, we speculated that cantharidin might also be able to fulfill its anticancer capacity in vivo as long as the proangiogenic side effect was abolished. Based on this hypothesis, we investigated whether the combination of cantharidin and antiangiogenic therapeutics could present additive anticancer effects in vivo. Ginsenoside Rg3 is one of the active ingredients of ginseng, and it can suppress tumor angiogenesis by impairing the biological activity of endothelial progenitor cells and attenuating VEGF-dependent p38/ERK signaling and Akt/endothelial nitric oxide synthase signaling in vitro^16,17^. Clinical studies have proven that Rg3 exhibited a good safety record and few side effects^18,19^. TAM is a non-steroidal antiestrogen drug frequently used in endocrine therapy and has been proven to be an inhibitor of the PKC pathway^11^, which could be the main pathway participating in the proangiogenic effect of cantharidin (Fig. 3). We previously showed that TAM could repress the growth of pancreatic cancer cells by inhibiting the PKC pathway^11^. Since both Rg3 and TAM present satisfactory tolerance in clinical practice and can inhibit pathways participating in proangiogenic effect of cantharidin, we investigated whether Rg3 and TAM could antagonize the growth-promoting effect of cantharidin in vivo.

**Fig. 4:**
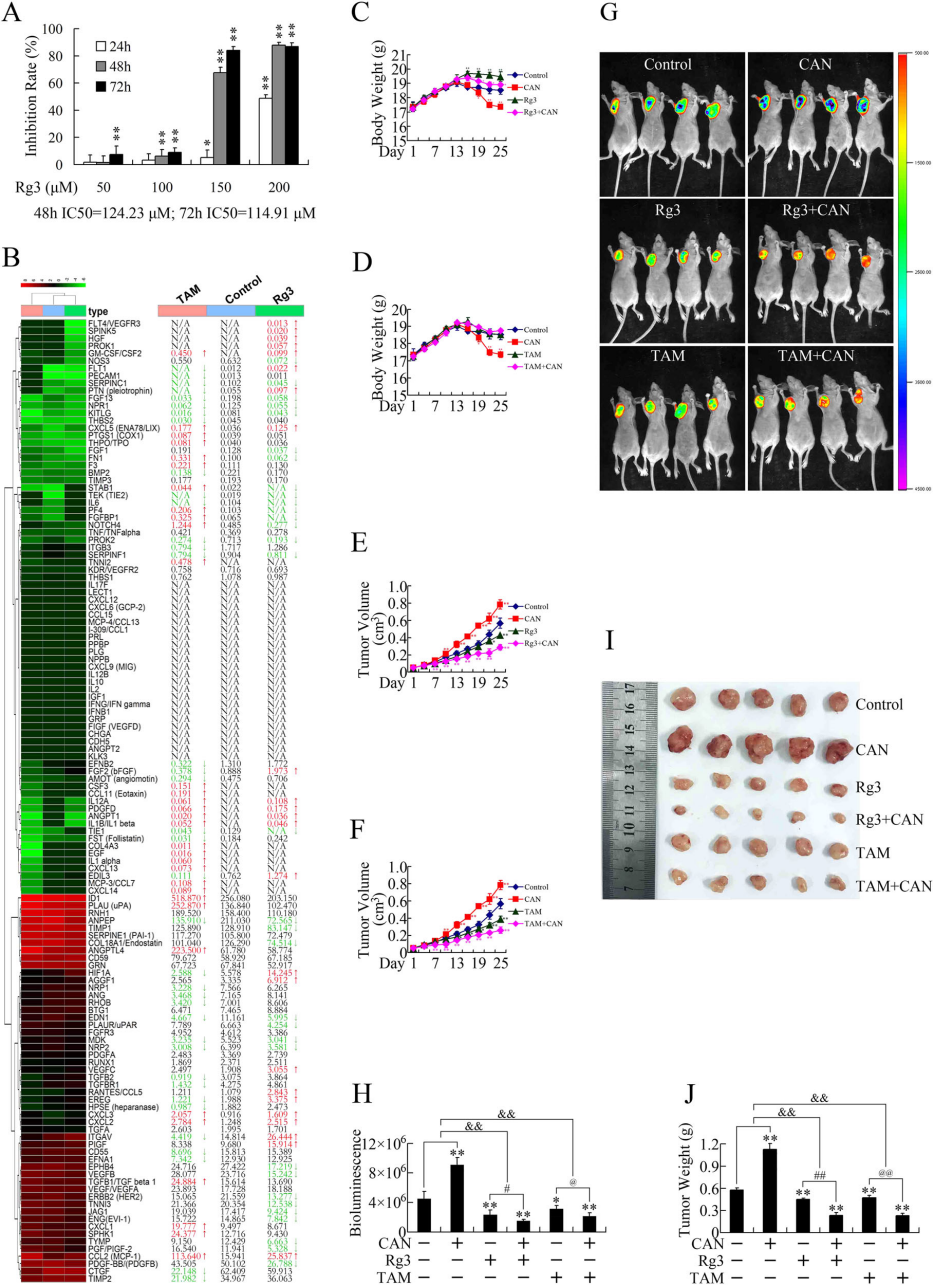
Both PKC-inhibiting tamoxifen and traditional medicine ingredient Rg3 attenuated the pro-growth effect of cantharidin in vivo. **a** Evaluation of the cytotoxicity of Rg3 in PANC-1 cells in vitro by MTT assay. Exposure to various concentrations of Rg3 inhibited PANC-1 cell growth in time- and dose-dependent manners. *P < 0.05 and **P < 0.01, significant differences vs. control group. **b** RNA-seq data of PANC-1 cells after treatment with tamoxifen (TAM) and Rg3. The up and down arrows indicate significantly upregulated or downregulated gene expression levels by 1.5-fold. **c**, **d** Body weight evaluations of subcutaneous xenograft models upon treatment with cantharidin and Rg3 (**c**) or TAM (**d**). **e**, **f** Tumor volume evaluations of subcutaneous xenografts after treated with cantharidin and Rg3 (**e**) or TAM (**f**). **g**, **h** Representative in vivo bioluminescent images (**g**) and bioluminescence analysis (**h**) showing the effects of cantharidin combined with Rg3 or TAM on pancreatic cancer subcutaneous xenografts. **i**, **j** Photographs of subcutaneous cancer xenografts (**i**) and tumor weight evaluation (**j**) of subjects treated by cantharidin combined with Rg3 or TAM. *P < 0.05 and **P < 0.01, significant differences vs. control group. ^#^*P* < 0.05, ^##^*P* < 0.01, ^@^*P* < 0.05, ^@@^*P* < 0.01, and ^&&^*P* < 0.01, significant differences between fold changes

As the cytotoxicity of Rg3 against pancreatic cancer cells had not been verified, MTT assays were first performed in vitro (Fig. 4a). As expected, Rg3 inhibits PANC-1 cell proliferation in both dose- and time-dependent manners. We then performed RNA-seq to obtain the gene expression profiles after treatment with Rg3 or TAM. As shown in Fig. 4b, although showing different patterns, both Rg3 and TAM repressed the expression of multiple angiogenesis-related genes. Moreover, both Rg3 and TAM reversed the growth-promoting effect of cantharidin and presented additive anticancer effects in combination with cantharidin (Fig. 4c–j).

### Antiangiogenic therapeutics antagonized the proangiogenic effect of cantharidin in vivo

**Fig. 5:**
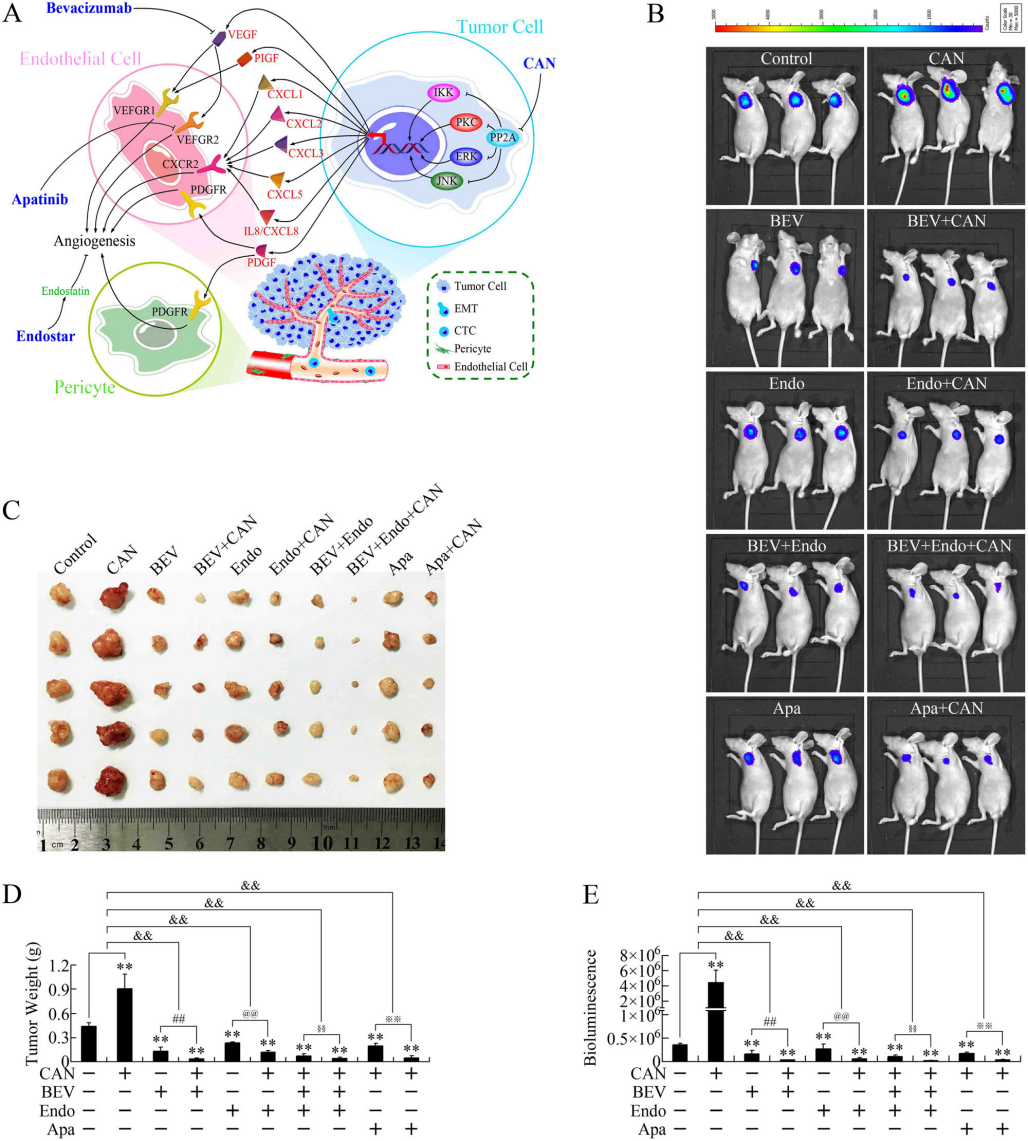
Antiangiogenic therapeutics impaired the pro-growth effect of cantharidin in vivo. **a** Potential mechanisms related to the proangiogenic effect of cantharidin. **b** Representative bioluminescent images showing the effects of cantharidin (CAN) and antiangiogenic therapeutics on pancreatic cancer subcutaneous xenografts. Three antiangiogenic therapeutics, including bevacizumab (BEV), Apatinib (Apa), and Endostar (Endo), were used. **c**–**e** Photograph (**c**), tumor weight (**d**), and bioluminescence (**e**) of subcutaneous xenografts. **P < 0.01, significant differences vs. control group. ^##^*P* < 0.01, ^@@^*P* < 0.01, ^§§^*P* < 0.01, and ^※※^*P* < 0.01, significant differences between groups. ^&&^*P* < 0.01, significant differences between fold changes

We then investigated whether specific antiangiogenic therapeutics could be more effective to constitute regimens with cantharidin. Three antiangiogenic therapeutics were used (Fig. 5a), including bevacizumab (a partially humanized monoclonal antibody targeting VEGF), Apatinib (an antiangiogenic agent targeting the intracellular ATP-binding site of VEGF receptor 2), and Endostar (a recombinant human endostatin).

As shown in Fig. 5b–e, when combined with bevacizumab, Apatinib, and/or Endostar, cantharidin repressed the growth of pancreatic cancer subcutaneous xenografts remarkably, indicating that cantharidin could play an effective anticancer role in vivo, as long as antiangiogenic therapeutics were applied. Notably, the combination of bevacizumab, Endostar, and cantharidin almost abolished the enlargement of xenografts, suggesting that this regimen holds promise for future clinical applications.

### Derivatives of cantharidin also presented in vivo growth-promoting effects that could be reversed by bevacizumab

**Fig. 6:**
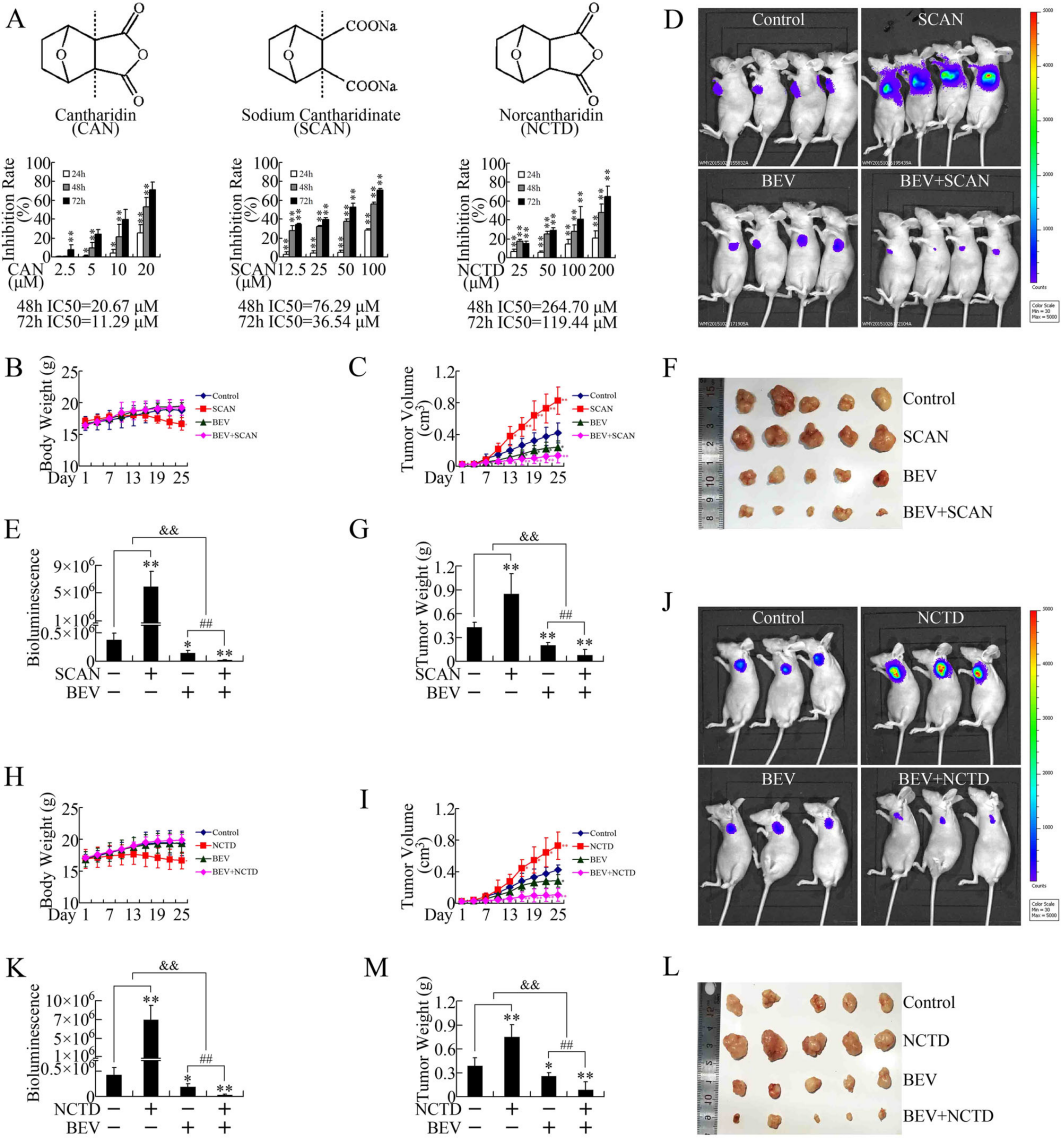
Bevacizumab attenuated the pro-growth effect of cantharidin derivatives in vivo. **a** Exposure to various concentrationsa of cantharidin (CAN), sodium cantharidinate (SCAN), and norcantharidin (NCTD) inhibited PANC-1 cell growth in dose- and time-dependent manners in vitro. **b**, **c** Body weight (**b**) and tumor volume (**c**) evaluations of subcutaneous xenograft models upon treatment with SCAN combined with bevacizumab (BEV). **d**, **e** Representative in vivo bioluminescent images (**d**) and bioluminescence analysis (**e**) showing the effects of SCAN combined with BEV on the growth of xenografts. **f**, **g** Photographs (**f**) and tumor weight evaluation (**g**) of xenografts treated by SCAN and/or BEV. **h**, **i** Body weight (**h**) and tumor volume (**i**) evaluations of subcutaneous xenograft models upon treatment with NCTD combined with BEV. **j**, **k** Representative in vivo bioluminescent images (**j**) and bioluminescence analysis (**k**) showing the effects of NCTD combined with BEV on the growth of xenografts. **l**, **m** Photographs (**l**) and tumor weight evaluation (**m**) of xenografts treated with NCTD and/or BEV. *P < 0.05 and **P < 0.01, significant differences vs. control group. ^##^*P* *<* 0.01, significant differences vs. BEV-treated groups. ^&&^*P* < 0.01, significant differences between fold changes

Studies have shown that cantharidin has significant side effects, such as nephrotoxicity. Therefore, several derivatives of cantharidin have been developed for clinical practice. The most widely used derivatives are sodium cantharidinate (SCAN) and norcantharidin (NCTD) (Fig. 6a). Considering the growth-promoting effect of cantharidin in vivo, it is necessary to evaluate whether the derivatives of cantharidin could also accelerate tumor growth. The cytotoxicities of SCAN and NCTD against pancreatic cancer cells were first verified in vitro using MTT assays. As expected, both SCAN and NCTD repressed the growth of pancreatic cancer cells in dose- and time-dependent manners, although the efficiencies of the derivatives were weaker than their precursor (Fig. 6a).

Consistent with cantharidin, both SCAN and NCTD promoted the growth of xenografts in vivo, which could be antagonized by bevacizumab (Fig. 6b–j). SCAN and NCTD are better tolerated than cantharidin; therefore, the combination of cantharidin derivatives and antiangiogenic therapeutics could represent more practical regimens.

Moreover, to better illustrate the roles played by cantharidin and its derivatives in solid tumors in vivo, we confirmed the antitumor effect of them in combination with antiangiogenic therapeutics in colorectal cancer subcutaneous xenografts models (Supplementary Figure 3). Compared with the control and single drug-treated groups, the tumors in the co-treated groups were much smaller, implying that cantharidin and its derivatives exhibited a significant antitumor effect in colorectal cancer when co-treated with antiangiogenic therapeutics.

## Discussion

Cantharidin, as a traditional antitumor drug, has been widely used in clinical practice in China. The antitumor activity of cantharidin, involving inhibiting migration, triggering apoptosis, arresting G2/M transition, and repressing invasion, has been explored in many in vitro studies^4–11^. However, conflicting with these antitumor roles, subcutaneous and orthotopic xenograft models clearly illustrated that cantharidin could accelerate the growth of pancreatic, lung, and colorectal cancers in vivo. In addition, the emergence and decay of the bioluminescence peak value suggested that cantharidin-treated xenografts might have a rich blood supply. Higher MVD^20^ was detected in cantharidin-treated xenografts, confirming the proangiogenic effect of cantharidin in vivo.

Increasing evidence shows that aberrant angiogenesis plays an essential role in tumor progression in vivo^19,21^. Abundant angiogenesis regulatory factors and oncogene activation are critical for supporting tumorigenic angiogenesis phenotypes^22^. In the present study, a microarray and an angiogenesis antibody array suggested that cantharidin significantly upregulated the expression levels of proangiogenic IL-8, GRO, VEGF, GM-CSF, and uPAR at both mRNA and protein levels. Besides, the Milliplex assay exhibited that cantharidin promoted the secretion of proangiogenic VEGF, IL-6, IL-8, and TNF-α. It is worth noting that cantharidin increased VEGF and IL-8 not only at the mRNA level but also at the protein and secretion levels. VEGF exerts a crucial role in promoting of vascular endothelial cell growth and is vital for the process of angiogenesis^23^. IL-8 is a pro-inflammatory cytokine that acts as a chemotactic agent. Besides, it is also a powerful angiogenic factor^24^, which contributes to the distant metastasis of cancer^25,26^. Therefore, VEGF and IL-8 may exert a major role in the proangiogenic effect of cantharidin.

Cantharidin is a potent and selective inhibitor of PP2A, a repressor of several oncogenic kinase pathways, including ERK, JNK, PKC, and NF-κB. Inhibition of PP2A can boost tumor growth by inducing phosphorylation and activating these oncogenic substrate kinases^27,28^. ERK and JNK are members of the mitogen-activated protein kinase family. Activation of ERK has been linked to cell survival, whereas JNK participates in proliferation^6^. PKC is a vital messenger for the transcriptional regulation of growth factor-responsive MMP genes, which promotes cancer cell invasion^29^. The NF-κB pathway is involved in the regulation of multiple genes participating in angiogenesis of pancreatic cancer^13^. In this study, inhibitors of these kinase pathways markedly attenuated the cantharidin-induced remodeling of the proangiogenic gene expression profiles, suggesting that all these pathways contribute to the provascularization effect of cantharidin. Notably, among the 25 investigated proangiogenic genes, the PKC inhibitor and NF-κB pathway inhibitor were the most effective reagents, attenuating the upregulation of 20 and 18 genes, respectively. This suggested that the PKC and NF-κB pathways could be the most relevant pathways participating in the proangiogenic effect of cantharidin.

TAM is a prototypical drug targeting estrogen receptor. Studies have revealed that TAM is a PKC inhibitor and can enhance the anti-pancreatic cancer effect of cantharidin by inhibiting the PKC signaling pathway in vitro^11^. In view of the significant role of PKC pathway in promoting angiogenesis of cantharidin, we evaluated whether TAM, as a PKC inhibitor, could antagonize the tumor-promoting effect of cantharidin. Ginsenoside Rg3, the predominant active constituent of ginseng, is a commonly used Chinese medicinal herb that inhibits biological activity of endothelial progenitor cells^16,17^. Furthermore, Rg3 has been reported to repress vascularization^18^. Therefore, we confirmed whether Rg3, as an antiangiogenic agent, could be used to antagonize the growth-promoting effect of cantharidin. As expected, cantharidin successfully fulfilled its antitumor potential in vivo in the presence of TAM or Rg3, presenting additive antitumor effects.

Clinical practices have proved that antiangiogenic therapy is an important and promising method for the treatment of cancer patients^30^. Researchers have made much effort to the direct discovery of new antiangiogenic drugs. Bevacizumab, Apatinib, and Endostar are all well-developed antiangiogenic agents that target VEGF, VEGFR-2, and endostatin, respectively, and have achieved considerable curative effect in patients with tumors^31–33^. Therefore, we evaluated the combination of cantharidin and these specific antiangiogenic therapeutics and found that these regimens presented remarkable additive antitumor effects.

Derivatives of cantharidin, such as SCAN and NCTD, have also been demonstrated to exert remarkable antitumor activity in vitro^9,34,35^. Consistent with the results for cantharidin, both SCAN and NCTD promoted tumor growth. In addition, the combination of SCAN or NCTD with bevacizumab inhibited the growth of pancreatic cancer subcutaneous xenografts dramatically.

Many reagents can repress cancer cell proliferation in vitro; however, in in vivo studies, the tumor microenvironment should be taken into consideration. Our present study revealed an unfavorable proangiogenic side effect of cantharidin via targeting the tumor angiogenic microenvironment in vivo. Antiangiogenic therapeutics or inhibitors of proangiogenic kinase pathways could antagonize the growth-promoting effect of cantharidin and present additive antitumor effects, exhibiting adequate efficacy. Therefore, rigorous preclinical investigations and clinical trials should be performed for any pharmaceuticals, even those derived from traditional medicine. We highly recommend that clinical applications of cantharidin and its derivatives should be performed on the premise of antivascularization therapy.

## Materials and methods

### Cell line and cultures

The human pancreatic cancer cell line PANC-1, the colorectal cancer cell line LoVo, and the lung cancer cell line NCI-H292 were all purchased from the American Type Culture Collection (ATCC; Manassas, VA, USA). PANC-1 and LoVo cells were maintained in Dulbecco’s modified medium (Gibco, Grand Island, NY, USA). NCI-H292 cells were maintained in Roswell Park Memorial Institute-1640 medium (Gibco). In all, 10% fetal calf serum (Gibco), 100 U/mL penicillin, and 100 mg/mL streptomycin were added to the medium. The atmosphere is humidified at 37 °C in a 5% CO_2_ incubator. Cells were tested for mycoplasma contamination and passaged every 2–3 days.

### Reagents

Cantharidin, OA, TAM, PD98059, SP600125, and GF109203X were purchased from Enzo Life Science International, Inc. (Plymouth Meeting, PA, USA). NCTD and EF-24 were purchased from Sigma (St. Louis, MO, USA). SCAN was purchased from Jinqiao Pharmaceutical Co. Ltd (Guizhou, China). Ginsenoside Rg3 was purchased from Shanghai Jinsui Bio-Technology Co. Ltd (Shanghai, China). Bevacizumab was purchased from Roche Pharma Co. Ltd (Basel, Switzerland). Endostar was purchased from Simcere-Medgenn Bio-pharmaceutical Co. Ltd (Shandong, China). Apatinib was purchased from Hengrui Medicine Co. Ltd (Jiangsu, China).

### Nude mouse tumor xenograft model and treatments

Four-week-old female BALB/c athymic nude mice were purchased from Shanghai SLAC Laboratory Animal Co. Ltd (Shanghai, China). According to the Soochow University Institutional Animal Care and Treatment Committee, all mice were given 1 week to adapt to the new environment and received humane care throughout our experiments.

For subcutaneous xenograft model, about 1 × 10^7^ cells in Matrigel (Becton Dickinson, Bedford, MA, USA) were injected together into the left side of the mice. Tumor volume was measured and recorded each day. For orthotopic pancreatic xenograft model, about 0.5 × 10^7^ cells stably expressing firefly luciferase in Matrigel were injected into the body tail of the pancreas of nude mice. After sample size estimation, all mice were grouped randomly. Investigators were blinded to the group allocation during the experiments.

Treatments were conducted according to the following methods: Cantharidin, SCAN, and NCTD, dissolved in dimethyl sulfoxide (DMSO), were administered every 3 days by intraperitoneal injection at doses of 1, 20, and 1.5 mg/kg, respectively. Bevacizumab (50 mg/kg every 3 days) and Endostar (50 mg/kg per day) were administered via the caudal vein. Apatinib, TAM, and Ginsenoside Rg3 were dissolved in DMSO and administered daily by intragastric administration at doses of 10, 0.5, and 20 mg/kg, respectively. For living imaging assay, the mice were anesthetized and then given 1.5 mg D-Luciferin of 100 µL phosphate-buffered serum (PBS) by intraperitoneal injection. Bioluminescence was imaged using an IVIS Lumina II apparatus (PerkinElmer, Waltham, MA, USA) or a Kodak In-Vivo Imaging System FX Pro (Rochester, NY, USA). When the experiments were terminated, the tumors were stripped and formalin-fixed for immunohistochemical assays.

### Immunohistochemistry

All resected tumor samples were fixed in 10% formalin and then embedded in paraffin by conventional treatment. Slices were cut into 4-μm thickness, heated for 30 min at 60 °C, and then dewaxed and hydrated. The slides were microwave treated for 5 min with an antigen repair buffer. After supplementing the buffer, microwave 5 min to heat the slides once again, then cool for 20 min. After blocking the endogenous peroxidase activity by adding 3% H_2_O_2_, the sections were incubated with 10% bovine serum albumin for 1 h to block non-specific binding. Rabbit anti-CD34 antibodies (ab81289; Abcam, London, UK) was used for immunohistochemical staining. A SuperPicture Polymer Detection Kit (No. 87–8963; Invitrogen, Carlsbad, CA, USA) was used to visualize the binding complex after incubating with the corresponding second antibody.

### Angiogenesis vascularity evaluation

MVD was defined as the number of blood vessels obtained by counting each field of view at the region of high vessel density. First, vascular endothelial cells were stained with anti-CD34 antibodies. The positive expression sites were brown or brownish yellow. The sections were placed under a low power microscope to determine the region of the highest staining density, defined as the “hot spot” area, and then placed under a 400-fold microscope to count the microvessels. Individual or clustered endothelial cells were counted. There were no scores for blood vessels in the peritumoral and necrotic areas. Cells that were unclear or fuzzy stained are not counted. A vascular lumen was not an essential structure for microvessel counts. The branching structure was calculated as one, except that vascular continuity was interrupted, in which case the branch structure was calculated as two different blood vessels. MVD was calculated as the average valve of blood vessel contours counted per mm^2^ from at least three hot spots.

### Milliplex assay

Secretion levels of IL-6, IL-8, TNF-α, and VEGF were detected using a multiplex biometric immunoassay (Cat. No. HCYTOMAG-60K; Millipore). Luminex Technology (Bio-Plex Workstation; Bio-Rad Laboratories, Hercules, CA, USA) was used to calculate the mean fluorescence intensity. Milliplex® Analyst 5.1 (Bio-Rad Laboratories) was applied for data analysis.

### Angiogenesis antibody array

Proteins were biotinylated in the samples and the superfluous biotin was removed by spin-filter. After dialyzing by PBS, biotinylated proteins were added to RayBio Human Angiogenesis Antibody Array membranes (RayBiotech, Norcross, GA, USA). By incubation with horseradish peroxidase–streptavidin, the captured biotinylated proteins could be measured. Then a chemiluminescence imaging system (RayBiotech) was applied to analyze the results. The internal control signals of each protein array chip were used for standardization. Quality control was performed by removing proteins with a signal intensity <50. A *t* test was used for the difference analysis, and fold changes ≥1.5 times were considered significant. Standardization was performed with internal control signals on each protein chip to remove proteins that were 50 times lower than the original signal intensity.

### Real-time PCR

Total RNA was first extracted by TRIzol reagent (Invitrogen) following its protocol. After quantification by spectrophotometry, reverse transcription was carried out in a system of 20 μL with 1 μg total RNA using a PrimeScript RT Kit (Takara, Otsu, Shiga, Japan) following the manufacturer’s protocol. Aliquots of cDNA were placed on a LightCycler 96 Real-time Quantitative PCR Detection system (Roche, Indianapolis, IN, USA) for PCR amplification. The reaction system was 25 μL, which consisted of the cDNA, forward and reverse primers, and SYBR Green PCR master mix (Roche). Three sets of duplicate wells were set for each sample, and the reaction was terminated after 40 cycles. The Ct value of each system was recorded. *B2M* gene was used as an internal standard. The relative transcription levels of mRNA were calculated by 2^−ΔCT^. Specific primers used in this study are listed in Table 1.

**Table 1:** Primers

Genes	Sense (5′–3′)	Antisense (5′–3′)	Product size (bp)
B2M	TCAAGAAGGTGGTGAAGCAG	AAGGTGGAGGAGTGGGTGTC	112
VEGFA	CACCCACCCACATACATACATTT	CCTCCCAACTCAAGTCCACAG	170
PGF/PIGF-2	AAGGGAGCTGCTGTCTGCG	CTTGCGGAGTCAGGAGCCCGTAGGT	192
PDGFA	ATTGTAGCACTCGGTGAAGCA	TCTGGAGTCGTTCCCAAAGC	247
CXCL1	CACCCCAAGAACATCCAAAGT	CCTTCAGGAACAGCCACCA	210
CXCL2	GCTTATTGGTGGCTGTTCCTG	ACACATTAGGCGCAATCCAG	101
CXCL3	GCCCAAACCGAAGTCATAGC	GAACCCTCGTAAGAAATAGTCAAAC	271
CXCL5	ACAGTGCCCTACGGTGGAAGT	CTCATCAAAGCAGGGAGTTCATA	266
IL-8/CXCL8	TGGATGGGTTCAGAGGCAC	GCAGGGCAGAAGGAATGGT	147
IL1α	TGACGACGCACTTGTAGCCAC	GCCAATGAAATGACTCCCTCT	111
IL10	TGGTGAAGGAGGATCGCTAGA	CCTTGATGTCTGGGTCTTGGTT	204
IL12A	TATGATGGCCCTGTGCCTTAG	CTATCAATAGTCACTGCCCGAAT	279
FIGF	CATCCCATCGGTCCACTAGGT	CAGCCACCACATCGGAACA	190
GM-CSF	ACACTGCTGCTGAGATGAATGA	AAAGGTGATAATCTGGGTTGCA	218
CSF3	CCTTCGCCTCTGCTTTCCA	CGTTCTGCTCTTCCCTGTCTTT	199
COL18A1	TCAGACCACGGCTCGATTTC	CTCAGCTCCCATTGCCTCA	154
EFNB2	GACGTCCAGAACTAGAAGCTGG	CACCAGCGTGATGATGATGACG	197
SPHK1	GAAGGGTGAGAAGGTGGAGG	TTATTTGGATTTGGTTCGTGGG	147
TIE1	CCAAGTACGTTGTGGAGGTGC	ACGGATGATGGTGCTTGTCTC	95
BTG1	CGGGTTACCGTTGTATTCGC	TTCGGCTGTCTACCATTTGC	246
CD55	GCTAAATTCTGCATCCCTCAAAC	TGTAACCTGGACGGCACTCAT	92
KDR	CCCAATAATCAGAGTGGCAGTG	CATAGACATAAATGACCGAGGCC	163
KITLG	TGTTGGATAAGCGAGATGGTAGT	TTCACGCACTCCACAAGGTC	146
PLAUR/uPAR	GCCGGGCTGTCACCTATT	CCACATCCAGGCACTGTTCTTC	132
RHOB	GCATCCAAGCCTACGACTACCT	CGGCCCTCATAGCACCTT	149
RUNX1	TGCCTCAGTGGAGACAAGTGG	GTCTCAGCCTGGTGAAAGCA	107

### MTT assay

MTT was performed to assess the ability of cell growth. Cells in logarithmic growth phase were adjusted to an appropriate cell density and seeded into 24-well culture plates. Twenty-four hours later, the cells were grouped and administered treatment according to the experimental requirements. After treatment, MTT and DMSO were added separately according to the protocol described in our previous study^6^. The absorbance value was measured at 490 nm by a microplate enzyme-linked immunosorbent assay reader (Bio-Rad Laboratories). Inhibition rate = [(mean control absorbance − mean experimental absorbance)/mean control absorbance] × 100 (%).

### RNA-seq analysis

First, total RNA was extracted using TRIzol reagent (Invitrogen) following its protocol. An RNeasy Mini Column (Qiagen, CA, USA) was then used to purify the extracted RNA. After quality evaluation, total RNA was sequenced and analyzed for mRNA expression profile (Novel Bioinformatics, Shanghai, China). We used FAST-QC to assess the original sequencing data and TopHat for RNA-seq alignment^36^. To identify the possible splicing junctions, MAQ was used to assemble the mapped reads. Besides, the unmapped reads also could define the splice junctions using the seed extension procedure. We performed the Limma algorithm to filter the genes after the analysis of significance and false discovery rate (FDR) according to the criteria: (i) fold change >1.5 or <0.67, (ii) FDR < 0.05^36^. Data has been uploaded to GEO database, the accession code of which is GSE114040.

### Statistical analysis

All experiments were conducted at least three times. The results were calculated as mean ± standard deviation. An unpaired Student’s *t* test was used for statistical analysis. An *F*-test to compare variances was performed, if necessary. *P* < 0.05 was considered statistically significant.

## Electronic supplementary material

Supplemental figure legends

Supplemental Figure 1

Supplemental Figure 2

Supplemental Figure 3
