# Supplementary material for: The combination of cantharidin and antiangiogenic therapeutics presents additive antitumor effects against pancreatic cancer
Source: Oncogenesis. 2018 Nov 26;7(11):94. doi: 10.1038/s41389-018-0102-2 (PMC6255842; doi:10.1038/s41389-018-0102-2)
Supplement: Supplementary file 1 — Supplemental figure legends [file 41389_2018_102_MOESM1_ESM.docx]

**Supplemental Figure Legends**

**Supplemental Figure 1. Effects of cantharidin on the growth of NCI-H292 lung cancer cells and LoVo colorectal cancer cells. (A-B)** Exposure to various concentrations of cantharidin resulted in dose- and time-dependent growth inhibition of NCI-H292 lung cancer cells (A) and LoVo colorectal cancer cells (B) *in vitro*. **(C-F)** Photographs (C) and tumor weight evaluation (E) of cantharidin (CAN)-treated subcutaneous xenografts of lung cell line NCI-H292. Photographs (D) and tumor weight evaluation (F) of CAN-treated subcutaneous xenografts of colorectal cancer cell line LoVo. **P* < 0.05 and ***P* < 0.01, significant differences *vs.* the respective control group.

**Supplemental Figure 2. Cantharidin promoted angiogenesis of lung and colorectal cancer xenografts.** Immunohistochemical examination of subcutaneous lung (A) and colorectal (B) cancer xenografts. Immunohistochemistry was performed using antibodies targeting CD34. Microvessel density (MVD) was determined according to the number of vessels per field, counted in the area of highest vascular density.

**Supplemental Figure 3. Anti-angiogenic therapeutics impaired the pro-growth effect of cantharidin and its derivatives on colorectal cancer subcutaneous xenografts *in vivo*. (A)** Photographs of subcutaneous cancer xenografts treated with cantharidin (CAN), sodium cantharidinate (SCAN), or norcantharidin (NCTD) in combination with Endostar (Endo) or bevacizumab (BEV). **(B-D)** Tumor weight evaluations of CAN (B), SCAN (C), or NCTD (D) combined with Endo. **(E-G)** Tumor weight evaluations of CAN (E), SCAN (F), or NCTD (F) combined with BEV. **P* < 0.05 and ***P* < 0.01, significant differences *vs.* control group. ^##^*P* < 0.01, significant differences *vs*. Endo- or BEV-treated groups. ^＆＆^*P* < 0.01, significant differences between fold changes.
