# Supplementary figures and images for: The combination of cantharidin and antiangiogenic therapeutics presents additive antitumor effects against pancreatic cancer
Source: Oncogenesis. 2018 Nov 26;7(11):94. doi: 10.1038/s41389-018-0102-2 (PMC6255842; doi:10.1038/s41389-018-0102-2)

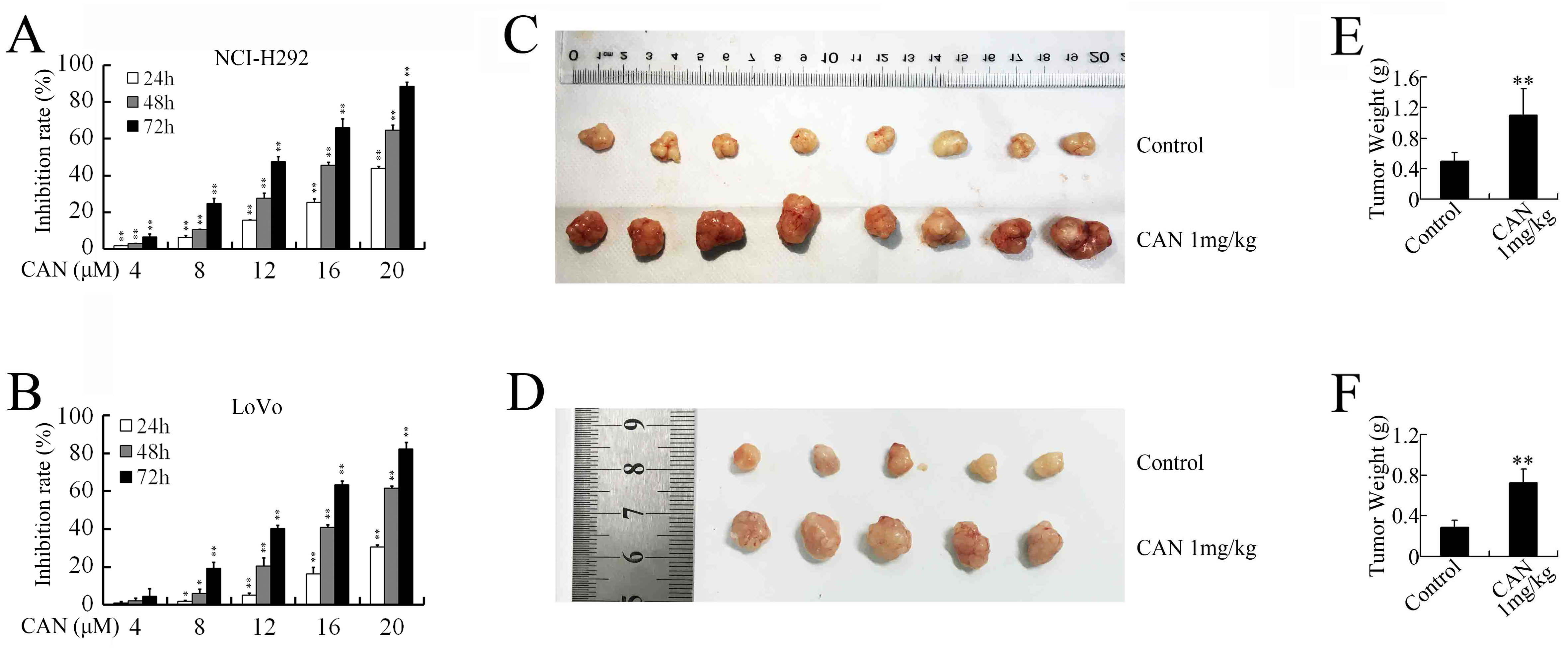

Supplement: Supplementary file 2 — Supplemental Figure 1 [file 41389_2018_102_MOESM2_ESM.tif]

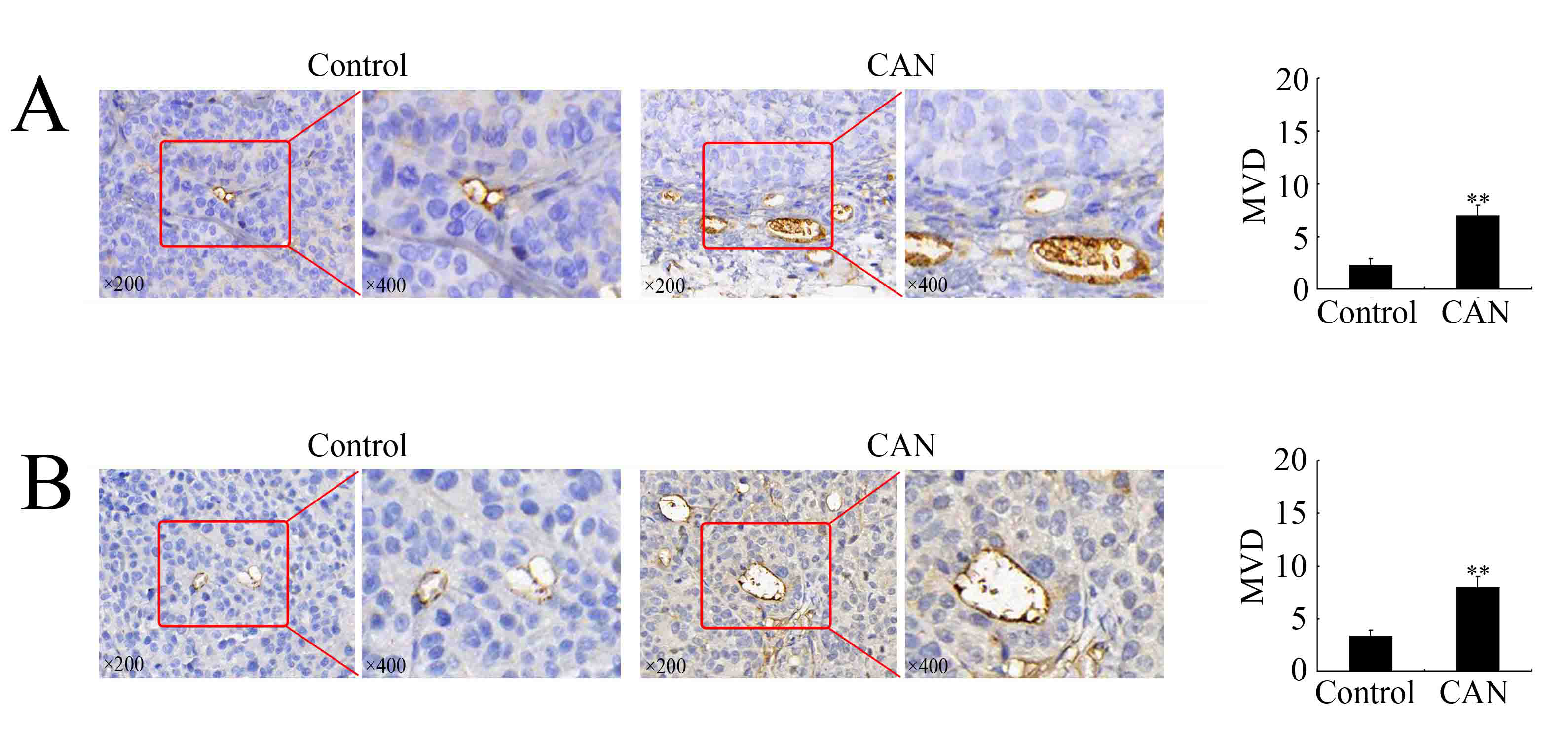

Supplement: Supplementary file 3 — Supplemental Figure 2 [file 41389_2018_102_MOESM3_ESM.tif]

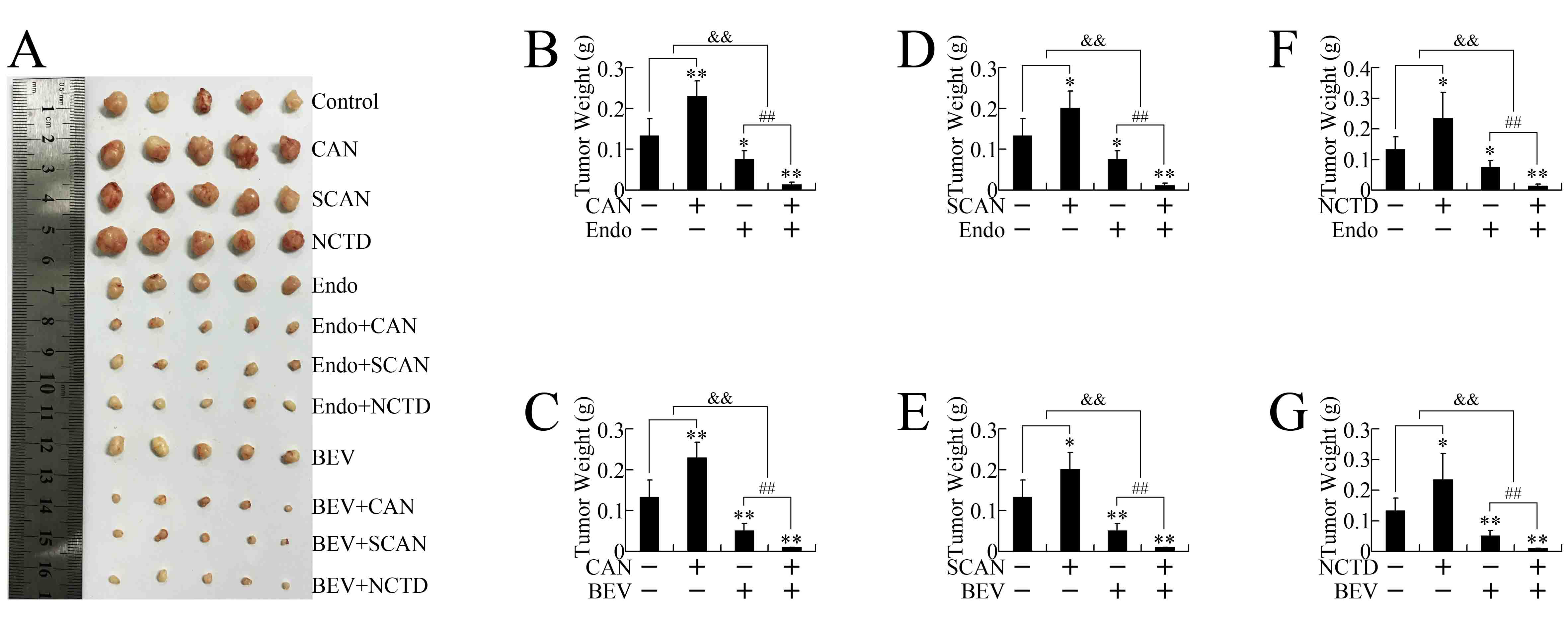

Supplement: Supplementary file 4 — Supplemental Figure 3 [file 41389_2018_102_MOESM4_ESM.tif]
